# On the verge of suicide from a suffering perspective

**DOI:** 10.1177/09697330261441779

**Published:** 2026-05-07

**Authors:** Anita Elvegaard, Dag Karterud, Jessica Hemberg

**Affiliations:** 1 Department of Health Sciences, Science and Engineering 1040 Åbo Akademi University, Vaasa, Finland; 2 Department of Nursing and Health Promotion, Faculty of Health Sciences, Oslo Metropolian University, Oslo, Norway

**Keywords:** literature review, empirical approaches, ethics of care/care ethics, theory/philosophical perspectives, suicide, caring, suffering

## Abstract

**Aim:**

To uncover the substance of how suffering is associated with human beings with thoughts or plans about ending their lives by suicide and to bring out more diversity, complexity, and contextuality of this phenomenon. Understanding as an important virtue underscores the safeguarding of the patient’s dignity as a holy element.

**Background:**

The study is grounded in a caring science perspective where suffering is seen as embodied in the human being. Methodology and design: This integrative literature review presents a combination of data from both theoretical and empirical literature with a reading of selected articles. Ethical ontological questions are prerequisites for highlighting caring understanding. Through the reading of a truthful written text of experienced suffering with love and compassion, it is possible to confirm and serve life and health.

**Data sources:**

Embase, CINAHL, PsycINFO, Scandinavian Journal of Caring Sciences, and Nursing Ethics. Ethical consideration: The study was conducted in accordance with the guidelines of the Finnish National Board on Research Integration.

**Results:**

Three distinct themes were identified as a basis for considerations related to suffering: (a) Suffering in time and space, (b) Bearable and unbearable suffering, and (c) Language as essential for being.

**Conclusion:**

The language of suffering in caring science is made visible. Caring understanding has an inherent ought to, embraced by ethos towards alleviation. Life-threatening suffering is revealed. Thinking of suicide as a way out can make it possible to endure the suffering. Lack of caring understanding was a threat to some of the patients’ dignity. Lack of evidence exists as to whether telling the suffering story is a movement in the direction of alleviation and this calls for further research to examine if caring and caring science can embrace this suffering.

## Introduction

Every year the number of suicides worldwide is estimated to be more than 720 000^1^ and the reversal of suicides as a global imperative is published by the WHO.^2^ Commitment across governments is needed from health and education work and the support of voluntary and statutory sectors. Academic institutions and schools, businesses, the industry, and the media also have important roles to play,^3^ to prevent suicide is everyone’s business.^4^ Many countries have introduced national suicide prevention strategies to show the government’s commitment,^5–7^ and different suicide prevention strategies have been presented over the years.^6,8–10^ The study targets individual human beings who have concrete thoughts, or plans about doing life-threatening self-harming or already have tried to take their own lives and survived^10^ and visualize close encounters with suicidality. Nomothetic research dominates the suicide prevention field,^11^ which has given us valuable background knowledge, but these insights do not reach the individual suffering human being. This study does not address the criticism of earlier research about having taken this field into a dead end of repetition research to find risk factors,^11,12^ but is striving for encountering diversity, complexity and contextuality^11^ with a strong relationship to the words used in the description of the suffering human being.

### Background

The first known description of suicidality is from approximately 2000 BC; a man suffered from life with a soul filled with horror and his longing for death ended in total annihilation.^13^ In antiquity, Aristotle (384–32 B.C.) meant that individuals who lacked self-control and voluntarily caused self-harm did an unjust action to themselves.^14^ The Bible describes suicides, among them Judas’s after the betrayal of Jesus, Judas went down and hanged himself (Matt 27.5).^15^ In modern times, psychache is identified as a deep pain and the main ingredient in suicide.^16^

Émile Durkheim (1897) emphasized the importance of context^11:130^ and pointed to an incompatible relationship between society and the group to which the individual belongs, in terms of being too weakly or too strongly integrated into the community^11:131^. Durkheim also claimed that suicide is not a distinct form of insanity.^17^ Later, there has been a strong opinion that suicide is a part of mental disease^11^; *mental pain can kill*.^18:113^ Nevertheless, disease is not a good enough explanation because most people with mental health diseases do not end their lives by suicide.^11^ Diseases causing significant bodily pain, like cancer are also associated with suicide.^7^ Actions are full of meaning^11^ and, therefore, we have to understand what suicidality is about for those concerned; what meaning suicidality has for them in their context.^11^ High levels of psychological stress and immense physical pain can make human beings in suicidal crises extremely vulnerable and ambivalent and may require professional care to mitigate suicidal thoughts.^3^

The WHO defines suicide as an act of deliberately killing oneself,^2^ but this definition can be problematized. People who really want to die, can survive an attempt. People who did not want to die may not survive because of unfortunate circumstances. People with alcohol or substance abuse problems or individuals in deep psychosis may not know what they are doing and do not fit into the definition that says that suicide is deliberate. Further, a person who once made a suicide attempt may never do it again. Or, a person who attempts suicide many times may 1 day complete the suicide. The totally unexpected suicide^11^ makes the complexity total. To speak of complexity is to make room for stories that can be sustaining for people.^11:300^

In caring science, promoting and protecting life is to confirm the human beings’ absolute dignity and holiness,^19^ which include their uniqueness,^20,21^ irreducibility,^20^ and irreplaceability.^22^ Their uniqueness has to do with the importance of the right to be oneself with one’s own distinctive characteristics realized in ways of expressing words and in body movements.^23^ The color of the unique human being’s expressions represents a core of integrity with a call to protect life.^24^ Irreducibility is associated with every human being as an entity of body, soul, and spirit and its core of integrity,^25^ which is embraced by an untouchable zone^23^ (Løgstrup) and has an appeal to protect the other’s vulnerability if necessary.^24^ Irreplaceability is closely related to the human being as fundamentally holy.^26^ Each single life has its own meaning and specific value, which is not indifferent to me.^24:10^ We are touched by the impression of the other, and Martinsen underscores the relevance of taking a few steps back to give the suffering other space, which, in turn, also protects the other’s holiness.^19^ These movements demand the use of a sensitive and tuned language that does not classify the human being but listens, wonders, and is open.^23:62^ This attention gives the language space to move^23^ close to the suffering human being and provides the opportunity to take care of the other’s uniqueness, irreducibility, and irreplaceability.

A suicide relieves a human being of intolerable pain, but this point of no return also leaves behind it a bridge of much suffering as difficult questions come to the fore about what was not seen or heard before the suicide. Therefore, the attempt to highlight suffering related to suicidality is a confirmation of these human beings’ distress and a striving for finding new insights that can be life-saving. An understanding of evidence as a search for truthfulness in written texts of patients’ experiences^27,28^ builds upon the view that suffering is a part of a human being’s life.^29–31^ If we deny the suffering, we also deny a part of our life and our possibilities to be a whole person.^32,18^

Suffering is seen as forces moving away from a state of well-being, a kind of harmony, to chaos that threatens this harmony.^29^ Negative forces gather our energy,^33^ and catch our attention according to the degree and strength of the suffering. At its worse the suffering is made visible as a real tragedy.^29^

Eriksson^32^ introduced having suffering as a kind of discomfort, and being in suffering as a sense of being threatened or feeling a sense of hopelessness. Becoming in suffering, is described as a struggle for something better, or the loss of something important or of a significant other, a movement between something positive in life and resolution.^32,34^ Everyday challenges including work-related stress are a kind of suffering of life^32^ that can turn out to be dangerous. Suffering of care^32^ or lack of care is an extra load to carry and a result of the absence of being seen, being met or being provided with needed help.^32^ A suicide reminds us of strong negative forces that got the upper hand and it is, therefore, critical to study suffering through the sufferers’ narrative descriptions.

## Aim

The aim is to uncover the substance of how suffering is related to human beings with thoughts or plans about taking their own lives by suicide and to bring out more diversity, complexity, and contextuality in the understanding of this phenomenon. Understanding as an important virtue underscores the safeguarding of the patient’s dignity as a holy element.

## Methodology

In order to more fully understand the phenomenon of suffering related to being near to take one’s own life, an integrative review, which combines theoretical and empirical data,^35,36^ is chosen. This includes a visualization of a range of studies by interrelating prior isolated phenomena and summarizing the findings to provide a more comprehensive understanding of suffering related^37^ to suicidality. To carry out a review from an explicit and theoretical perspective is, according to Kirkevold,^37^ of importance because it leads to the possibility of reading, analyzing, and presenting the selected articles in relation to this perspective.

### Theory as a basis for a caring understanding of suffering

The theories represented by Kari Martinsen and Katie Eriksson^25^ constitute the fundamental basis for the considerations^37^ related to the essence of suffering. Martinsen has her background in a phenomenological and historical perspective.^28:14^ She first worked with the philosophy of Heidegger, but has for many years concentrated on Løgstrup’s texts.^25:125^ Eriksson is educated in a conceptual analytic tradition and in Gadamer’s hermeneutic.^28:14^ Both Martinsen and Eriksson view the human being as a unit of body, soul, and spirit and caring as based on ethos, love, and compassion,^28^ which constitutes the soul of the understanding of care and qualifies a caring understanding. The harmony at the core of ethos is challenged when something is difficult, uncomfortable, or painful, such as by the impression of the other who is close to take their own life. We get to know the other’s vulnerability through the reading of a text of unhearable suffering such as anxiety, chronic pain, troubled thinking, and remembering.^38^ All these have ethical appeals about being recognized^39^ and are, therefore, not neutral but embraced by the ethical ontological questions^25^ below. The questions are prerequisites for being able to attain a caring understanding in a suicidal crisis when we face the unpredictability and the deep suffering associated with the issue of suicide. Martinsen asks *what is the calling in the other’s calling?*,^24^:14,^28^:65 and emphasizes the senses as an opening to this understanding. *To see is also related to listening*, *it is rather to listen than to see* 28:19. In this, we have in common that we are delivered to each other and related to one another as fellow human beings. This ontological dependency^22,40–44^ calls for an ought to, an ethical preparedness with a demand of taking care of my fellow human beings.^24^

The question, *what is good caring?* asked by Eriksson,^45:25^ is a vision^46^ that envisions a safeguarding of the individual patient’s dignity^25^ as a sacred dimension that represents the soundboard in this perspective.^46^

### Caring understanding as a basis for the visualization of suffering

The question *what is good caring*^45:25^ can be transformed into *what is good caring for the visualization of suffering* which is strongly connected with the ultimate ethical demand, the avoidance of hurting anybody.^46^ This demand is meant to confirm the human being’s absolute dignity and holiness,^19^ a protection of the *untouchable zone,* an ethical warning area in which we should not intervene.^19,47^ The ethical implication is here related to the disclosure of the individual suffering text, with a warning about a description that excludes the other or takes away the sufferer’s own description as the *speaking I*.^48^ This warning is justified by the view that it is in each unique painful story we find the secret source of every alleviation of suffering, either in a natural self-caring process, or together with a caring other. The implication of this is, on the other hand, that a visualization of deep suffering without an openness to caring, leaves the suffering human being behind^39^ with open wounds which is a visualization of suffering that disqualifies caring science. It is also in the individual suffering story we find that the sources of beautiful experiences sprout to life and liberation^44,46^ which also contributes to qualify a good caring understanding and a good caring visualization of suffering.

### The philosophical basis for the collection, reading, and interpretation of the data

A phenomenological and hermeneutic approach founded by Husserl^49,50^ and Heidegger and further developed by Gadamer^51^ and Ricoeur^41^ is chosen for the collection, reading, and interpretation of the relevant material on the basis of caring science theory.

For Husserl it was important to highlight the Case Itself as it is and to allow recognition to be subjected to the limits of the visualization,^50^ here of the phenomenon of suffering. The awareness of one’s pre-understanding is stated by Heidegger as something from which we cannot free ourselves^40^ in the search for a meaningful understanding, according to Gadamer.^51^ The pre-understanding influences the interpretation of the suffering text, but in turn, is also changed and enlightened by the interaction^52^ with the essence in the empirical material. Husserl, however, put pre-understanding in parentheses. He emphasized consciousness more than knowledge as an act where the other is made present to me as it is revealed,^53^ different from the objective, outer world, and with a willingness to take a step back to obtain space to receive the other, instead of conquering and capturing.^39^ A conscious attitude is possible which at the same time includes both pre-understanding, the influence of personal experiences from private life, work, and theoretical background,^52^ and bracketing setting aside previous thoughts and mental barriers along the horizons of our thinking.^52^ To give these positions the same status as unifying contradictions—which is a description taken from Løgstrup^54^ and which is transferable in this context as sources in the striving for a caring understanding, colors this process; we pay attention to the presence of the other through our senses, being touched as human beings.^39^ Simultaneously, already gained experiences, attitudes, and judgments are included together with the willingness to become impressed and touched by the new reading, embraced by the ultimate ethical demand which is the avoidance of hurting the other.^46^ Understanding as an important virtue^55,56^ undergirds the safeguarding of the other’s dignity as a holy element in the serving of their life and health. As an inherent quality in a caring understanding, dignity is justified as something we should show respect for and honor.^20,26^

### The reading process

**Figure 1.**
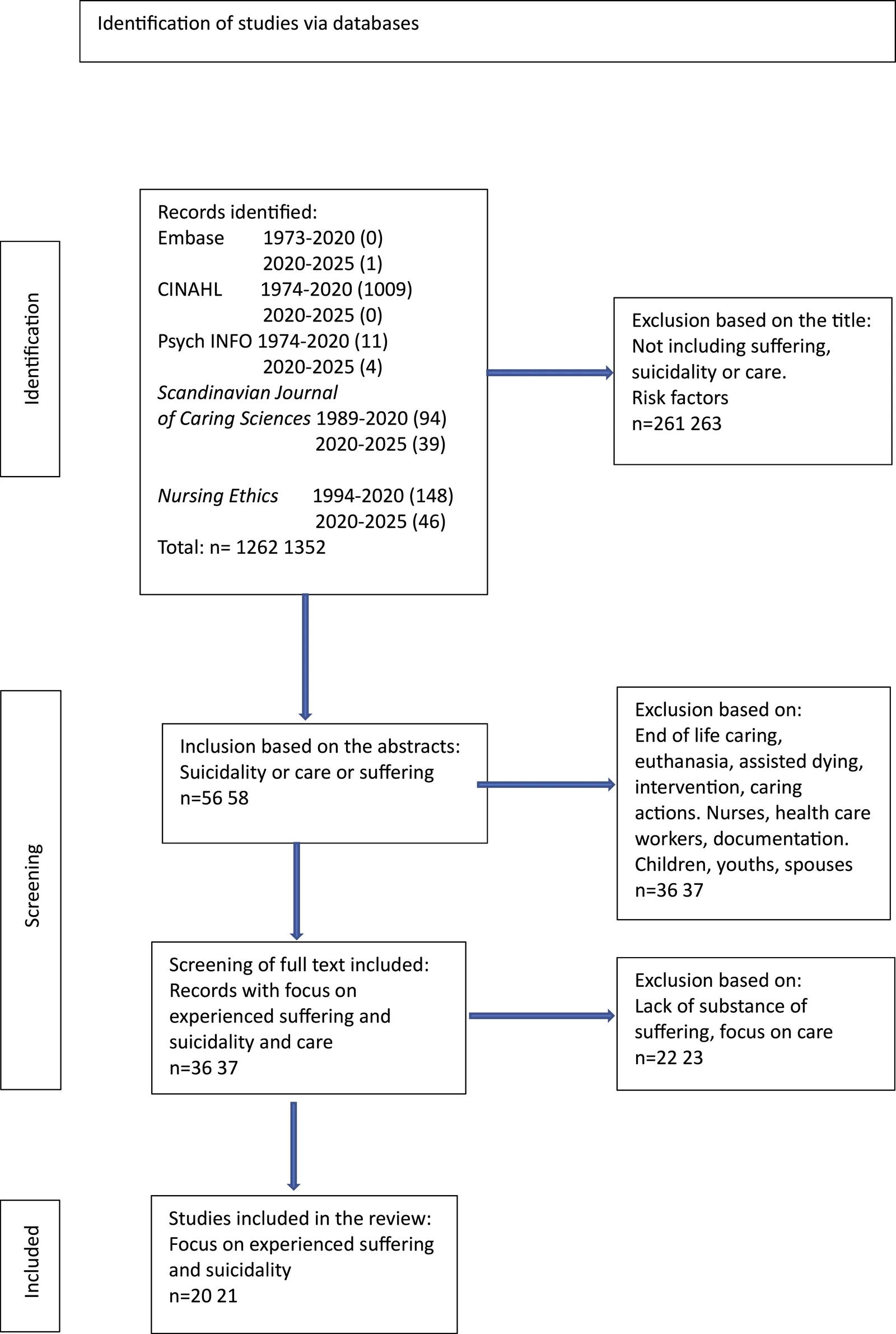
Overview of the selection process.

*A searching for truth, an attempt to uncover truth and live in it* is ethics in its deepest sense and represents the work of starting to see, listen, and know.^55^ Reading is a way of being in the world which constitutes our existence.^42^ In this process, we find a hidden possibility to recognize the text in an authentic way if we focus on the main theme without being disturbed by whims or locked in by prejudgment (same). According to Eriksson,^46^ to truly understand a text means to learn to read it and then decipher its meaning and deeper motive. The sound of the language relates to suffering and is an articulation of what has already been there by means of speech.^41^ Reading will also open up for different sounds to a listening connected to a promise in the thinking process,^33^ and in the question of the substance of suffering as it is revealed in the chosen articles. The encounter with the empirical data from the selected material in the search process, shown in Figure 1, resulted in an overview that includes words, phrases, and sentences about an individuals’ suffering related to suicidality. The suffering text here comes to the fore without any interpretation of it.^52^

From this immature visualization of the data, the process made movements into a more interpretative understanding; *to interpret is to follow the path of thought opened up by the text, to place oneself…towards the orientation of the text*.^52^ In this study, the analysis at this stage refers to the description of suffering in the selected articles synthesized with the chosen theory, which is a process that ended up with three different main themes; *Suffering in time and space, Bearable and unbearable suffering as a source of protection and life-threatening suffering, and Language is essential for being*.

The process subsequently moved into a more in-depth understanding. Ricoeur used the term *hermeneutic arc*^52^ to describe the movement back and forth between the parts of the text and a view of the whole during this step. He did not discount the hermeneutic circle as proposed by Heidegger and Gadamer, but underlined the process between explanation and understanding.^52^ The acts of this interpretation are also part of different areas of knowledge. First, the experiences and beliefs that the researcher brings to the task include spoken words and their pre-understanding, which is documented. The second type of contribution is the researcher’s knowledge and experiences^52^ of suffering individuals near to taking their own lives. The interpreter’s progress to a deeper understanding of the patients’ experiences demonstrates how the interpreter in this process eventually achieves an ever deeper understanding and this is highlighted in the subthemes. At this stage, the process is to some degree influenced by the reader’s understanding of the meaning of the individuals’ suffering texts.

### Presentation

The process of interpreting the text ideally creates in the interpreter a new understanding^52^ of suffering related to suicidality. According to Ricoeur,^52^ the interpretation of a text culminates in the self-interpretation of a subject who then understands themselves better, differently, or simply begins to understand themselves.^52^ Knowing oneself is the emerging of a new self, a hermeneutics of *I am,* rather than *I think*.^52^ The practical implications of this are twofold; if the researcher comes to know self in a new way, it will be possible to “be new,^52^ with a novel pre-understanding in the encounter with new suffering texts. Secondly, it can be possible to interact with a new suffering text in new ways,^52^ in caring science this always means in the direction of alleviation. This is, for instance, highlighted as connected to one of the main themes of this study: *Suffering in time and space.* The questions are here raised about a caring understanding and what it is to be seen and understood as related to the findings in Hultsjö et al.’s study.^57^ The importance of giving patients in similar situations in the future other caring answers activates the two main questions in this study; *what is calling in the suffering human being’s calling*^24^ and *what is good caring*^45^ *(understanding)?* These questions can form the basis for building up another *arc* (Ricoeur), another ethical preparedness, tuned or retuned in neighborly love^25:128^ and compassion towards alleviation. The consequences of the movements that are initiated also open up a connection to what Ricoeur called *Living communication*,^52^ which refers to the process between explanation and understanding. Ricoeur also explained the relationship between explaining and interpretation; *to explain is to bring out structure that is the internal relations of dependence which constitute the statics of the text. To interpret is to follow the path of thought opened up by the text, to place oneself…towards the orientation of the text*.^52^ In this light, the subthemes that evolved in this research project are presented in the subheadings. The interpreter’s progress from a naïve to an in-depth understanding of the patients’ experiences demonstrates how a process of moving back and forth between explanations and understandings eventually leads to an in-depth understanding.

The articulated words return to silence in a space of freedom^33^ where reflection can breathe in a rhythm of a conscious way of reaching a new insight. Thinking back and forth, the inclusion of memories, and what can be possible as well as the realization in this moment are all aspects of this working process. In every moment, the language has the potential to exceed^33^; to bring a new understanding, another understanding or to confirm an already understood meaning of the written text. Nothing is evident before it is expressed in words,^28,41^ and the essence of this language is a subjective line of work.^33^ *Something is made visible as being*.^33:217^

### Design

In May 2020, the first data search was carried out in Embase, CINAHL, Psych INFO, *Scandinavian Journal of Caring Sciences*, and *Nursing Ethics* with the key words *Experience of suffering and suicide and caring*. Table 1 displays the inclusion and exclusion criteria. The second search was conducted in the same digital sources in February 2026.

**Table 1.** Inclusion and exclusion criteria were chosen in an effort to conduct the research among adults over 18 to secure a more homogeneous group and make it easier to identify clearer and more consistent patterns in the data.

Inclusions	Exclusions
Primary sources 1974–2025	
Nursing and caring context	
Adults, age over 18	Children and adolescents
Suffering perspective	Focus on risk factors
Experience of suffering	
Individual level	System level, group level, caregivers, caring
Suicide prevention	Voluntary assisted dying, euthanasia
Before suicide	After suicide

For an overview of the selection process, see Figure 1.

The identification of titles after the search with key words resulted in 1262 articles in the first search and 1352 articles in the second search. The titles with risk factors were excluded, and so were titles without suicidality or care or suicide prevention, which left 261 titles the first time and 263 the second time. The screening of the abstracts included care or suffering related to suicide prevention and resulted in 56 articles in 2020 and 58 articles in 2026.

The next exclusions were based on caring, intervention, end–of-life caring, assisted dying, euthanasia, health care workers, children, youths, spouses, or documentation, which left 36 and 37 articles, respectively. These were all screened in full text, and after having found a lack of the substance of suffering and experienced suffering related to suicidality, it resulted in 22 and 23 articles. The last inclusion was based on focus on experienced suffering and suicidality which resulted in the 20 and 21 studies that were included in the present study. Among the selected articles, 13 applied qualitative approaches,^57–69^ 3 used quantitative methods,^70–72^ and 5 implemented mixed approaches.^73–77^ The different approaches affect the influence on how language is used, chosen words, and by which they are formed. This means that some of the articles fall outside the language associated with caring science, but here the considerations of the substance of suffering are chosen to be of importance, and hence essential for not isolating caring science research in the striving for the understanding of suffering in this context. It also underscores the importance of crossing boundaries between different suicide prevention fields and to promote the caring for what Ricoeur^52^ called a *living communication* that here reflects the language used in the suicide prevention field. A negative consequence of this choice is a reduced interpretative rigor^52^ related to a less accurate representation of the understanding within the framework and worldview in this research, but on the other hand, it also shows a conscious awareness and consideration of the view on suffering. The selected articles are given the same attention, including direct quotes, which also supports the aim of the study providing the opportunities to reveal a broader understanding^52^ of suffering related to suicidality. A living communication^52^ deepens the relationship to the text and the understanding.

## Findings

Three distinct themes were identified as a basis for considerations related to suffering associated with suicidality: (a) Suffering in time and space, (b) Bearable and unbearable suffering, between suffering as a source of protection and life-threatening suffering, and (c) Language as essential for being. These are described in the following, also with subthemes presented as subheadings focused on providing in-depth-knowledge.

### Suffering in time and space

Suffering is a movement in time and space.^31,39,54,78^ The consciousness of life and death is inherent in our relation to time.^79^ Ontological time is independent of the human being, heads forward, never comes back and ends up with inevitable natural death.^19^ Experienced time is split into past, present, and future. With the opportunity to recall the irrevocable when grief needs to be filled up with good memories and wishes for the future, we get in touch with a hope for something good or better. Both positive reminiscence and hope are life-confirming spacious concepts which affect the experience of time.^44^

Yet, in more than half of the selected studies hopelessness seems to be very difficult to handle and was in most of them related to *suicidality*.^57–65,70,73–75,77^ The feeling of *hopelessness and also helplessness underlie all other emotions associated with suicide*.^11:136^ A female patient was overloaded at work. Pushed into a too tight corner in life, she was overwhelmed by distressed feelings and the suicide death was the only solution she could think of.^58^ These soundless inner movements stretched out their qualities in the room as a fundamental dimension^79^ with an impact on how the appreciation of her health and suffering is shaped and vice versa.

#### When something of importance is lost

A male patient felt imprisoned and experienced stagnation after the death of his spouse causing repeated suicide attempts. Another male patient became sad and depressed with a feeling of personal failure after an ended relationship. He also found it difficult to be without work, to be poor, and to be in an insecure housing situation. He withdrew from others and ended up making a suicide attempt.^58^ Suicide attempts among older women were explained in Silva et al.’s study (2018) through their experiences of a meaningless life associated with a sense of a loss of value for their closest and dearest.^77^ A female respondent^59^ described thoughts of suicide; …*I managed somehow hour by hour, but then… I became totally apathetic;* the important life force was lost.

A male’s growing loss of economic safety threatened the possibility to carry on his business, pay salaries to the employees, and take care of the economic duties in the family. He saw no way out. He experienced sadness, insomnia, loss of appetite, irritability, hopelessness, and 3 months of feeling depressed resulted in suicide ideation becoming a constant companion for him as help and relief.^60^

Among men with economic responsibility in a family, financial strain culminated in anger, protest, and sadness, and an increase in suicide ideation. Lack of support and irritation from their spouses were parts of their burdens. Pessimism was related to having given up the belief that the economy could improve again in the future.^71^ Among patients with psychosis, losing contact with an important other was a critical experience before they took their own lives.^74^ Loss of coherence in the sense of self was found related to suicidality in a study made by Benson et al. (2013)^77^ Cumulative losses around social bounds were stated to be central to older men’s depression, apathy for living, and thought about suicide (Oliffe, 2011).^77^

#### Having no home or feeling homeless in life

Stressful life events were described among women in a homeless situation. Hopelessness, low self-esteem, social isolation, and great difficulties in achieving employment and reintegration revealed a difficult life situation where almost half of them had attempted suicide.^75^ Skodlar et al. (2018)^77^ tried to understand the complexities of suicidal tendencies in patients with schizophrenia. The patients’ feelings of solitude and inferiority and their inability to relate to others were found to be connected with a closeness to life-threatening behavior.^77^ Before the patients died by suicide, having no home were related to a feeling of shame and being excluded.^74^ A low degree of belonging and a sense of being a burden to others had a strong relationship to suicidality and some of the patients had lost the belief that someone or something could help them.^65^ Other patients in the same situation had the feeling that nobody cared for them (Moore, 1997).^77^ Not being understood or acknowledged and feeling burdensome reinforced negative self-appraisals and triggered suicide thoughts among people with bipolar disorders (Owen et al., 2015).^77^

A patient in Richards et al.’s study^76^ did not want to leave the home after having been asked about suicidality; *everybody just freaks out and wants to get you hospitalized… it doesn´t make me feel better*^76^ *…*, a refusal to be punished to give away the freedom which jeopardized the feeling of safety connected to being at home in life.

Feelings of being ignored and mistreated in the health care system after an accident gave a female patient^58^ a sense of being homeless in life and thoughts about committing suicide. Another patient had the feelings of *being homeless with no strength left* related *to emptiness, meaninglessness, hopelessness, worthlessness, shame and guilt…*and that *life ended here and that the future did not exist…*^58^

Feeling socially disconnected and experiencing identity stigma provided for feeling suicidal (Ali and Gibson, 2019)^77^ and suffering from internalized stigma, contributed to suicidal ideation in Bautista et al.’s study (2017).^77^

#### Long-lasting suffering and having or losing the reason for staying alive

A sense of personal failure together with worthlessness were found in some of the studies,^59,63,64,66^ as well as *self-loathing*.^57^ Threats to dignity,^59,67^ especially lasting threats, take away natural strength and can result in emotional fragility and frustration.^59^ Exploring lived experiences of suicidality among civilians with severe traumatic brain injury (TBI), Knight et al. (2020) identified a sense of loss of self, hidden disabilities, a need to rely on others, and the use of unhelpful self-reliance, which all were associated with suicidality.^77^ In a study of patients diagnosed with depression in multiple sclerosis (Gaskill et al., 2011),^77^ the researchers found loss of control in life, increased family tension, and loss of perceived masculinity or femininity, and also hopelessness and loneliness related to suicidality.

Three months preceding their suicide deaths,^74^ 20 of 21 patients with psychosis had one or more negative critical experiences related to physical illness, loss of contact with an important other, being impoverished, and loss of home. In most cases it appeared to be a culmination of prolonged suffering, lack of meaningful and daily occupation, and suicidal deliberations. Ten of the 21 reported having expressed deep despair and hopelessness during the last 4 weeks before the suicide.^74^

Lakeman and FitzGerald^73^ conducted their research among patients who lived with suicidality. Young people’s struggle against giving in to suicide did not diminish with time. Existential crises and emotional distress were experienced as overwhelming terror. Finding a new meaningful interest gave content in life, and reinforced their decision to keep on living.^73^

Suffering related to serious psychiatric disease, loss of energy, friends, a job, and of fellowship, included feelings of shame. Discussing the right to live was related to life-supporting want as a common fate. Longing for someone to love was limited by the feeling of sickness as an obstacle. Their interests in different forms of art were in touch with natural needs of searching joy-of-life activities.^67^ Will and belief in something were movements towards betterment and effected in relationships in Vatne and Nåden’s study.^65^ Dialogue and cooperation created safety and the ability to cope with suffering, and hence hope and the will to struggle for life.^65^ Earlier stages in life affected the self-view, such as problems and breaks with important people, the death of a mother, substance abuse, physical injuries, excessive work-load, and loss of meaningful tasks. It was also described that bullying, identity problems and parents’ divorce… *started the blues… I can remember the anxiety…*when *thoughts about suicide came creeping in my teens.* A deep impact on their adulthood resulted in feelings of shame and guilt; ...*I still have some of these thoughts, that it is my fault*… *there was so much negativity that I somehow found no meaning in anything, then I felt I was worth nothing at all…* Before the suicide attempt, they described… *being dragged down by uncomfortable forces… being in a dark room… cannot find the door; not finding the light switch*.^64^

Major negative impact to health in adulthood with developed PTSD was described among men after experiences of sexual violation in the early stages of life. Hopelessness, lack of self-esteem and loneliness, and thoughts about the best way to die from self-injury with fatal outcome were a part of everyday life.^63^

Hultsjö et al.^57^ investigated patients’ experiences before they took their own lives during ongoing care. Emotional difficulties included despair, alienation, and self-loathing together with wishes to be seen and understood. Taking their legacy seriously is to take responsibility for the glimpse of hope for a life worth living for those in similar difficult situations in the future. The relevant questions to listen to are: what is a good caring understanding of despair, alienation, and self-loathing, and what is it to be seen and understood? These questions have their place in a room we are sharing with one another.^80^

### Bearable and unbearable suffering, between suffering as a source of protection and a threat to life

Suffering is visible through moving movements between bearable and unbearable,^39^ and this is seen in several of the selected articles.^57,60,61,63,66–68,72,73^

#### Bearable suffering as a source of protection

Bearable suffering is embraced by a zest for life, desire, and satisfaction and informs us when something is threatening and is therefore a source of protection. Humans strive for balance and harmony^32^; anxiety seeks safety, sadness tries to reach joy and happiness. Sorrow longs for reunion and love, anger is a defense against anxiety, guilt is related to responsibility and reconciliation and shame call for inner peace.

Suffering can also be a prerequisite for personal development^81^ and contribute to mental and emotional maturity.^29^ It reminds us to never forget individual power and possibilities for coming back to a more normal life after health is or has been threatened. Meaning in life had a protective role against severe suicidal intention in some of the studies.^67,72,73^ Even though feeling worthless, a patient described *the value of living*^64^ which is a visualization nurturing the touch of belief in a better life.

Becoming aware of the desire to live and the experience of connectedness was understood as pertaining to the patient’s inner resources and having someone who cares in the aftermath of suicide attempts. Encounters with health personnel were found to be alleviating if the patients were treated with respect and kindness.^65^ Longing for a better life among adults who had lived through suffering and regained health was found by Hemberg (2015).^59^ A male patient’s depression, social isolation, and sense of personal failure were also an opening to hope for a better life after he had told his suffering story, including his thoughts about suicide.^73^ Hultsjö explained that patients’ longing for being seen and understood before the suicide was an opening to hope for a better life that also is worth living.^57^ Hope is a part of health and a source of the bearable part of suffering as opposed to the unbearable. As long as a human being close to die by suicide is still alive, hope is also alive regardless if it is only a tiny little straw held up by the warmth of the pulse rhythm.

#### Life-threatening suffering

When suffering turns out to be unbearable, it affects both ontological and spiritual levels^61^ and can even pose a threat to life, meaning it dominates our thoughts, feelings, and acts and suicide death can be seen as a relief, and as the only option left when negative emotions become unbearable.^57,60^ But thinking about suicide can also be consoling^11,60,68^ because it makes it possible to endure the suffering without acting in a self-destructive way.

The unbearable is formulated as deep pain^9^; pressure, frustration, despair, anxiety, and anger; alienation and loneliness, sadness, grief, rejection, self-loathing, and shame.^57–61,64–68,77^

When the struggle for a good life has ceased^57,59,63,64,67,73,75^ the suffering at its worst is an inside chaos of stress and anxiety with lost hope.^59^ Some patients almost lost their zest for life due to aggrieved dignity …*great pain and anxiety… deep darkness and hopelessness just made me want to get out of there.* Self-destructive forces can make one’s own judgment out of tune,^63^ restrict intellectual focus,^66^ or lead to *frozen thoughts*^7^ and result in the idea of cessation^66^*…thoughts of suicide emerged in my worst state of suffering (female*).^59^

Contact with one’s own dignity^72^ can be replaced with low self-esteem,^62,75^ and feelings of not being worth anything^64,66,74^ or being a burden to others,^17^ all of these are associated with suicidality. Not being seen or understood by others, or being ignored seems to be a deep form of life suffering.^58,65,73^ Severe suicide intent was identified among advanced cancer patients in relation to greater pain intensity, disheartenment, helplessness, a sense of failure, and hopelessness.^72^

Patients experiencing first episode psychosis had intense emotional reactions, confusion, disturbed thoughts, and perceptions. Function disruption could result in further burdens, depression, and suicide intent, fear of mental deterioration, hopelessness, low self-esteem, and social isolation. As many as 40% of them reported suicidal ideation and 31% suicide attempts.^62^ Nilsson et al.’s study uncovered a male patient’s torturing hallucinations …*I heard voices and got this delusion that everyone in the city had committed suicide, and there was only me left, I thought I also had to commit suicide*.^67^ Before the suicide attempt the patients communicated a feeling of being dragged down by uncomfortable forces.^64^ Hultsjö^8^ found that the suicide death was the only solution to banish the darkness, and hopelessness and despair were found to be a total blackness in Rehnsfeldt and Eriksson’s study.^61^

Unbearable suffering is explained as ending up with destructive actions or suicide attempts in some of the studies.^58,60,63,70,73^ Among women in a homeless situation,^75^ 46% had attempted suicide. Physical and sexual abuse during childhood, impaired and neglectful parenting had serious consequences and were important vulnerability factors for the suicide attempts. The women suffered from high levels of physical and mental health problems and described sexual and physical abuse and intimate partner violence. Depression, fearfulness and low self-esteem were among the feelings that were strongly associated with their suicide attempts. The patients in Lakeman and FitzGerald’s study^73^ explained the suicide attempts was an escape in desperation and helplessness. Fitzpatrick^58^ found that grief and lost hope, imprisonment, and rejection ended in three suicide attempts for a male patient.

Feelings of being ignored and mistreated after an accident gave a female patient frustrations, insufferable pain, powerlessness, and hopelessness and a belief that nobody could help which all led to a suicide attempt. A relationship ended and a male patient’s sadness and depression resulted in social isolation and a sense of personal failure after unemployment. Ongoing financial problems, poverty, and insecure finances culminated in a suicide attempt.^73^

Experiencing marked irritability, depression, rumination, anxiety, sleep disturbances, and withdrawing from social gatherings, and with facets of impulsivity could potentially cause an individual to engage in suicide behaviors.^70^

### Language is essential for being

*Suffering lacks a specific language, but in its infinite silence there are forms of expressions that we can perceive with our innermost and sensitive movements, our mutuality and our compassion*.^31:2^ Every human being is a secret source for a text^82^ with a meaningful richness^71^ and to give something a word or a name means that this something has existence, language is *the house of being, it is coming to us, it hits us* and it *changes us*.^33:151,158^

Someone with suicidal intention will often try to struggle and manage the psychache with a desperate inner conversation. Existential pain and meaninglessness are weighed against escape possibilities or thinkable solutions.^83^ Language has a unique ability to organize and reorganize human beings’ experiences and can thus, through a basic self-organized activity such as self-caring, contribute to more stability or increased differentiation, and in this way make the overwhelming feelings more manageable.

Beskow et al. found that positive experiences can dissolve dysfunctional inner thought patterns with the creation of important words.^83^ On the other hand, being hit by overwhelming difficulties can lead to a broken verbal communication as described by Fleischer.^83^ Combined with hopelessness it can result in a suicide plan.^66^

#### A suffering text with an ethical appeal to a caring consciousness tuned in the direction of alleviation

A caring consciousness gives life to and protects the human being’s suffering text within the given promise^28^ about using a language that is listening to the patients’ experienced suffering,^20,23,55,84,85^ also when it is more or less hidden. Every human being has their own value in their own reality and is his own master,^55^ or is her own queen.

To have a relationship with the human body’s language^39^ of unarticulated thoughts and more or less unheard emotions is especially important when encountering unbearable suffering which, according to Eriksson, can be wordless.^32^ An often-silent darkness was found among the patients before they took their own lives.^57^ This silence is a source of concern and is connected to what Martinsen expresses as being touched by and have a feeling of compassion.^24^ A strong self-destructive force among male survivors who had been exposed to sexual abuse permeated unexpressed and unbearable suffering and appeared in life-threatening behaviors. Driving too fast and without seatbelts, drinking and the consumption of other drugs to kill emotional pain made them feel bad and worthless. Shattered self-esteem, shame, and loneliness characterized their lives. They associated physical pain and other health problems with the violence to which they had been exposed. Avoiding frequent negative thinking resulted in a longing for finding inner peace and developed into thoughts about the best way to end their lives through suicide.^63^

The encounter with these men could easily have been dominated by a relationship to their most visible and hearable destructive actions and neglecting their silent and deep suffering instead of taking responsibility for the inner worrying language. But this would have been to step out of the caring perspective and to ignore their *language as the house of being*^33:158^ and so leaving them alone in deep suffering. Caring is always a move in the direction of alleviation and when it is not, it is *lack of caring*^32:12^ and then it is also something we do outside of the house of ethos.

#### Telling their suffering story made it worse or reduced suicidality

Richard et al.^76^ found fear related to disclosing suicide intent before a suicide attempt, anticipating stigma and overreaction from the helpers and being afraid of losing autonomy related to hospitalization. In Tarrier’s study among patients suffering from a first episode of psychosis related to trauma and suicidal behavior,^62^ 28 of 35 patients indicated they were traumatized by being hospitalized. Major reasons were *being confused, scared following police insensitivity, adverse staff attitudes, and being forced to take medication.*

Vatne and Nåden^64^ observed in a study by Wiklander that *patients’ shame after a suicide attempt increased when health personnel responded with negative attitudes*. The negative attitudes reveal an understanding in the wrong direction. These patients’ suffering texts are a calling for a listening reader and must be tuned or retuned toward alleviation. At this moment, a caring understanding is strongly associated with Martinsen’s ethical ontological question; *what is calling in the other’s calling?*^24^ This calling for an answer in caring science is embraced by Eriksson’s question; *what is good caring?*^45^ The question represents an appeal to take responsibility, to be in the world and search for truth, an attempt to uncover the truth and live in it,^55^ a constitution of our existence.^42^ A willingness to take responsibility for these suffering human beings’ texts, in a good way, is also a willingness to protect life as well as to safeguard the patients’ dignity and the hidden sources of beautiful experiences sprout to life and liberation.^44,46^

In Fitzpatrick’s study,^5^ *where* the patients recently had made a nonfatal suicide attempt, one of the males experienced that telling his story opened up for a better life. Another male patient revealed that his grief, lost hope, imprisonment, and decay related to the death of his spouse were mixed up with a disclosure of what he needed in order to flourish. The patients in Trygvadottir et al.’s study^63^ found it very difficult to find words to express their suicidal suffering, but when they opened up a high level of positive energy followed the disclosure. When the human being can read their text, interpret themselves, they may have attained an understanding as close as it is possible to achieve,^46^ and it can be possible *through philological work to restore what is lost or hurt*.^55:72^

## Discussion

This study uncovers complexity and broadens the understanding of suffering in relation to human beings with thoughts or plans about ending their lives, which is important because of the demands put on the concepts and language that should be used when serving these human beings. A unique patient in imminent danger of taking his or her own life is calling for being treated as an interpreting subject^48^ and this study is therefore highlighting the suffering language. The reason for having made this choice is, for instance, related to the unique human being’s separation from the concept *being suicidal,* because in caring science the human being is never a part of a diagnostic description. Caring for the other’s dignity is to express that the human being is someone *with* suicidal thoughts or someone who is *having* suicidal plans. Thus, when describing the other’s situation, also when the pain is deep, it is a part of the unit human being and not vice versa. Firstly, this confirmation of absolute dignity and holiness^19^ presents the human being as unique,^20,21^ irreducible^20^ and irreplaceable.^22^ Secondly, holiness is also something we recognize with our senses, something we should take care of, an *untouchable zone,* an ethical warning area in which we should not intervene.^19,47^

The problem is that we are already *inside* of this zone in the encounter with the other in this coherence and our carefulness should not prevent us from describing the vulnerability of others, which would have been to leave them alone when they are in need of help. Neither does it mean taking over the other’s description of suffering, because then we would invade the other’s self-understanding. Here we are faced with complexity and dilemmas in the striving for a chosen caring understanding intended as a movement towards relief.

Løgstrup suggests a way to handle these conflicts. The *untouchable zone* is not only a warning area, it is also a protection, an unseen and unheard conscious line of tension between unifying oppositions.^47^ What qualifies the unifying oppositions is their grounding in caring science’s house of ethos, which protects life and human dignity. Understanding that bears the mark of either carelessness or sentimentality-represents in two extremes-and is thus outside this area. It also disqualifies a living communication held up by the tension between the unifying oppositions, which can be essential in the protection of the other’s life and health. In this striving, the position of awareness and acknowledgment about our limited possibility means to have a clear idea that it is impossible to fully understand the other,^86^ who is calling for an unsure openness to something we cannot have in our power. This openness gives space to the other who is capable to understand self. On the other hand, a decision has to be made related to a life-saving insight that can be held up by an attitude of the simultaneousness in the ethical ontological question: *does my understanding help or does it hurt the other?* Without a continuous relationship to this question, the tension in the zone of untouchability will lose its power and can turn into either an objective senseless description or a subjective or private presentation. Both positions are outside a caring understanding and the caring home of ethos that is grounded in an embrace of the suffering human being. Thus, the reading of the untouchable zone can be understood as a responsibility to take care of the presence of the tension therein as a basis for good caring understanding.

From this perspective, critical questions can be raised to research that visualize deep fragility; when suffering appears as naked facts it affects the reader, like the nakedness Levinas refers to in the close encounter with the other’s face; *as responsibility for the other, met me as a face*,^22:95^ an access he describes as *straight away ethical*.^46:85^ Jonas declares this as a responsibility we can resist but not deny.^43^ The call in the individual’s cries of pain is possible to resist if we only take the outside position and at its worst let their suffering entertain us from a distance. Yet, their fates and difficulties with surviving deep suffering tell their true stories that we cannot deny.

An understanding of suffering is not enough and highlights the difference between understanding and caring understanding because what qualifies the latter is that an inherent ought to, embraced by ethos, wishes the other a better life situation.^46^ *The saying is a way of greeting the* Other*, but to greet the Other is already to answer for him*.^22:88^ This is calling for an authenticity as a force that also binds us to a course of action.

A caring understanding in this study, is meant to move in the direction of alleviation, and it is not about relief *per se,* but highlights suffering that is not overshadowed by a too quick response based upon our need to help: it confirms the patient’s inner difficulties and provides a needed space for suffering. The language that belongs here is expressions that resound with the other.^28^

The evidence is, therefore, related to a reading or listening to the suffering text that underscores the safeguarding of the patient’s dignity as a holy element, as something powerful that we should respect. Separated from the concept “suicidal,” the human being receives validity independently from what they are doing, or in which state they are at the moment, and exemplifies the idea of the human being’s unqualified dignity.^39^

As opposed to the concept “being suicidal,” the formulation “to be near to taking one’s own life,” represents the closeness to a potentially life-threatening action together with the distance from a possible destructive action. The human being’s existence is thus given its own unique space and confirms them as fundamentally holy^87^ which is to take an ethical position that accepts life and rejects suicide. This is not a rejection to talk about suicide thoughts or plans which in itself can be preventive^6,9,21,28,29,58,63,65,68,69,72,73,83^ because it makes it possible to endure the suffering related to the comfort in the knowing that there can be a way out.

When the words are dressed in ethical affiliations that take care of the other, ethics comes before ontology.^88^ Through this way of using language, we get access to the other as an experiencing and interpreting subject, as a form of reflective humility, which challenges us to use sensibility, analytic and synthetic abilities as well as professional assessments.^48^ This non-instrumentalist knowledge about ethics, communication, and understanding of a context is a basis for dealing with particularity and its relationship with universality. In the nature of particularity lies an inherent openness to the contradiction of the word, which denotes the universality sphere when we recognize something which is common. Human consciousness makes it possible through memories of our own experience to understand each other and includes the knowing of what suffering and pain is.

To isolate the particular is a mistake where the subject is circling around itself with a missing relatedness to others in corresponding situations. This means we do not fulfill the process of coming in contact with the truth; instead, we create a possible pitfall. Openness between a single situation and many other similar situations will protect the phenomenon from superficial subjectivism.^89^ The presentation is then trustworthy^12^ and so qualifies the approach as valid. *Being is embraced in the truth. Even if the truth is considered as never definite, there is a promise of a more complete and an adequate truth*.^22:91^ Another pitfall is the description of the other as an object if we close the description of the other and deny the other as “a speaking I.”^48^ This distancing of the other as an object facilitates the possibility for manipulation, control, and inhumanity (same). To keep the other as a subject and as a fellow human being protects against inhumanity. According to Ekeland,^48^ the implication for this has to be that ethics has to become before the evidence. *I speak of responsibility as the essential, primary and fundamental structure of subjectivity. I describe subjectivity in ethical terms*.^22:95^

Describing the suffering human being as a *risk patient* will be an epistemological mistake, and also an ethical transgression that makes the patient the victim of this understanding,^48^ and is to take a position outside of and beyond caring understanding. The opposite is to use words to confirm the unique other so it can be possible to recognize the description and for the words to reach inner difficulties, searching a place with possibilities, to find support and comfort.

The selected studies reveal unbearable suffering as life threatening,^57,60,61,64–68,73^ with a deep impact on thoughts, emotions and actions.

This means that something of what is seen as most important in relation to life and health is broken. The experience of not feeling home in life excludes an individual from time, in relation to other human beings and is a silent call from the tunnel vision,^3^ for the *inner ought to* 46:21–33, and is also a call for further studies with the focus on good caring, about alleviation *per se* for suffering human beings who are on the verge of ending their lives by suicide.

## Conclusion

The identification of suffering with the use of a language that recognizes a human being with thoughts, intents, or concrete plans about suicide is rooted in the ideographic sphere where the other is taken care of as a unique subject. Firstly, the human being as holy is associated with the confirmation of absolute dignity and presents the human being as unique, irreducible, and irreplaceable. Secondly, holiness is something we recognize with our senses and should take care of, which in this connection refers to the *untouchable zone* (Løgstrup); an ethical warning area. The zone is housing a tension between *unifying oppositions*,^47^ a tension that is maintained by the ethical ontological question; *do my understanding help or does it hurt the other?* A lack of caring understanding was found to be a threat to some of the patients’ dignity and confirmed the opposite of relief. The calling in the other’s calling is found to be *a caring understanding* with awareness of the complexity and unpredictability of suicidality. Caring understanding with its inherent *ought to* is embraced by ethos towards alleviation. A good caring visualization of suffering is confirmed to be a striving for the safeguarding of the individual patient’s dignity. Unbearable suffering is revealed as life threatening, but is also disclosed as preventive because it makes it possible to endure the suffering associated with the comfort in the knowing that there can be a way out. Unpredictability is found in one of the studies where the patients had made suicide attempts after having reported no suicide ideation.

## Supplemental material

Supplemental material - On the verge of suicide from a suffering perspective Supplemental material for On the verge of suicide from a suffering perspective by Anita Elvegaard, Dag Karterud, Jessica Hemberg in Nursing Ethics.
