# Supplementary material for: On the verge of suicide from a suffering perspective
Source: Nurs Ethics. 2026 May 7;33(6):1735–54. doi: 10.1177/09697330261441779 (PMC13522427; doi:10.1177/09697330261441779)
Supplement: Supplementary file 1 [file sj-pdf-1-nej-10.1177_09697330261441779.pdf]

Studies included in the review

Authors (year)	Country	Methodology/Design	Participants	Aims	Substance of suffering
1. Costanza et al. (2020)	Switzerland	A case- control study and descriptive statistical analyses.	70 patients and 70 controls were enrolled from March 2018- November 2019.	Investigate the relationship between demoralization (D), meaning in life (MIL), suicide ideation (SI) and suicide behavior (SB) in patients with chronic pain.	The patients suffered from neuropathic pain, lower back pain, fibromyalgia or joint pain. Greater pain intensity was associated with the presence of D and functional disability among inpatients independently of illness severity. Loss of sense of dignity. Severe SI was identified. MIL has a protective role toward SI and SB. Negative correlation was found between MIL and SI and SB among older adults, veterans, and patients affected by psychiatric disorders.
2. Economou et al. (2013)	Greece	Cross-sectional telephone surveys were conducted in 2009 and 2011.	A sample of 2192 and 2256.	Self-reported measure described changes in suicidal ideation (SI) and suicide attempts (SA) during economic crises.	People with major depression reported SA 97 times more than others. Experiencing financial strain resulted in anger, sadness and pessimism about the future. The spouses interacted in a non-supportive and irritable manner. A recent SA was reported 53% more among married than unmarried. People with a previous history of SA were 14 times more likely to report a recent SA than those without. An increase in the prevalence of SI was observed among men from 2009-2011, but not among women.
3. Fitzpatrick (2014)	Australia	A narrative inquiry.<br>.	4 persons from two community health centers who were receiving crises and management services were included.	How persons who had recently acted a nonfatal SA accounted for their actions through telling stories.	The death of a spouse (William) caused three times SA. Grief, lost hope, mute, imprisonment and decay were mixed up with love and confession as revelatory and articulation of what he needed to truly flourish. Being overloaded at work (Beth), despair, hopelessness, and diminished choices ended with thoughts about SA. An accident caused insufferable pain (Anita) and feelings of being ignored and mistreated within the health care and lost hope led to SA**.** She also had valuable insight. Ended relationship (Michael) caused sadness, withdrawing from others, depression, isolation and a sense of personal failure. Together with unemployment, poverty and insecure housing it all culminated in SA. Telling his story opened up the hope for a better life.
4. Fredriksson & Lindström (2002)	Finland	Hermeneutic design. Caring conversations Naive reading. Structural analysis and critical reflection.	20 patients at an outpatient unit participated.	How psychiatric patients in conversation with nurses narrate their experiences of suffering.	Four facades revealed: the alone and strong, the victim, the invisible, the resigned. Fear, a feeling of being abandoned or deserted and a feeling of being disliked and not wanted. This in turn invoked rage, hatred and revenge. Evilness experienced both inwards and outwards towards others. The facade was a help to cope with suffering and shame. A want was to regain the pride**.** A freely narrating can either hide or reveal suffering and then regaining vital part of oneself.
5. Hemberg (2017)	Finland	Hermeneutic approach. Interviews.	10 adults	To expand the understanding of suffering. Suffering as described by the human beings themselves.	The pain takes over everything. No joy, deep darkness, an inside chaos of stress and anxiety. Feelings of sinking physically and being chased by wolves. Lack of resting, taking sleeping pills, wanting to sleep, lost hope and horror for what might be hidden underneath could end up with a question of surviving. Feelings of being emotionally stunned. Thoughts of suicide emerged in the worst state of suffering. *I managed somehow hour by hour but then… I became totally apathetic. I just lay in my bed* (Female age 61). *Great unbearable pain and anxiety… deep darkness, and hopelessness just made me want to get out of there* (Female age 24). *Being ...homeless in yourself... with… no strength left…related to emptiness, meaninglessness, hopelessness, worthlessness, shame and guilt. I had the feeling that life …ended here and that the future did not exist...I saw myself alone out in a storm in the middle of a dark night. It was a struggle for life* (Female 61). A sense of guilt were related to the feeling of being weaker than others... *the shame is something I will never get rid of (Female 41).* This might have no facial expressions, instead the person can pretend to be happy. Some almost lost their zest for life due to aggrieved dignity. Longing for health and better life might also be hidden underneath.

Studies included in the review

Authors (year)	Country	Methodology/Design	Participants	Aims	Substance of suffering
6. Hultsjö et al. (2019)	Sweden	Phenomenological perspective. Reflective Life World research.	12 interviews with mental health professionals were conducted.	To explore experiences in regard to circumstances that cause the patient to take their own life during ongoing care.	The patients had difficulties dealing with despair, alienation, shame and self-loathing. They described the life as meaningless, empty and hopeless, but they wanted to be seen and understood. They had lost something important which not could be realized. Their darkness was often silent and wordless. To take their own life was seen as a relief from unbearable suffering and only solution to banish the darkness. It was a borderline between life and death, small life changes could cause giving up the struggle.
7. Lakeman and FitzGerald (2008)	Australia	A systematically qualitative review and thematic content analysis.	12 studies were identified.	How people live with suicidality or recover a desire to live.	Psychache is deep pain and suffering and can take over every aspect of people´s life. Young peoples´ struggling against giving in to suicide did not diminish with time. Men with HIV experienced overwhelming terror and emotional distress. Longing for closeness, desiring connectedness and struggling to open up, breaking into outer dialogue are found. Among older adults both meaning and meaninglessness were described. Overt SI was found among widowers, and ambivalence to living and carelessness. To keep on living was related to the discovering of a new interest. Widowed men were encapsulated in stoicism or not giving in. Being engulfed in pain and suffering and depression were identified by the lack of caring and frustration with loss of self-deciding. SA was an escape in desperation and helplessness and a try to become less visible to others and resulted in loneliness and isolation. Ambivalence about suicide was found. Both SI and SA had a symbolic function as ´rock bottom´ experience that could change the meaning and purpose to a new commitment in life.
8. Nilsson et al. (2008)	Norway	Hermeneutic approach. A descriptive and explorative design. Participatory observation and interviews in the patients´ homes.	8 patients participated.	The purpose is to present the results of a study of the significance of loneliness in patients with serious psychiatric disease.	Suffering owing to illness resulted in loss of energy, friends, a job. Loss of fellowship was an intense suffering with a feeling of shame. All wanted to have someone to love, but stated their sickness as an obstacle. To live alone was something good with the liberty to lock and unlock their own door but they did not feel their homes as real homes. Some expressed existential anxiety and inner strangeness. One had torturing hallucinations…*I heard voices and got this delusion that everyone in the city had committed suicide, and there was only me left. I thought I also had to commit suicide.* Suffering was connected to care when being forced to take medication or experiencing prejudiced attitude. Want as a common fate became visible and was related to discussions about the right to live. Their interests in different forms of art can be seen as a health potential.
9. Pompili (2019a)	Italy	A case report.	A 63- years old man	How to understand suicidality.	Problems with carrying out his business, paying salaries to his employees, and to support his family resulted in sadness, insomnia, loss of appetite, irritability and hopelessness. He felt he was in a tunnel and saw no way out. Things got worse when the crise eroded his saved money. Unimaginable physical suffering with unpleasant sensations in the abdomen was related to anxiety. After months of feeling depressed, SI became the companion that could provide help and relief. He was both contemplating suicide and attached to life activities. Before he decided to kill himself, he contacted a friend.
10. Pompili (2019 b)	Italy	An essay	Literature study	A broader understanding of the suicidal mind.	Main sources of this pain are shame, anger, loneliness, despair and frustration over denied needs (Shneidman). Constriction is defined as tunnel vision with dichotomous thinking. Signals are given of being tired of living, thinking about death and a want to die. Hopelessness after experiencing being unable to communicate the suffering to the people assigned to help could end up with a suicide plan trying to avoid any interference of others.
11. Rehnsfeldt & Eriksson (2004)	Sweden	Hermeneutic approach. Letter-writing between the informants and the researcher. Interviews.	6 patients participated.	To understand suffering in relation to the encounter between the suffering person and the caregiver.	By expressing suffering, spiritual needs for meaning in life can be called forth from the patient´s inner world. The need to express oneself emerges when it is accepted and the abyss presents itself on a conscious level. It is a struggle between conscious openness and confronting and between paradoxes like hope-hopelessness, and the encounter as an opportunity to discover or create meaning. The patient is longing for genuine compassion.

Studies included in the review

Authors (year)	Country	Methodology/Design	Participants	Aims	Substance of suffering
12. Richards et al. (2019)	USA	Electronic health record data were used to identify the patients. Qualitative interviews. Deductive and inductive content analysis.	26 patients who made a suicide attempt (SA) within the last two months after reporting having no suicide ideation (SI) participated.	Factors that may facilitate or preclude patients from disclosing suicide ideation (SI).	They who reported no thoughts of self-harm did not experience SI at the time of the screening or feared the outcome of disclosure, including stigma, overreaction, and loss of autonomy. The patients used alcohol at the time of the SA or after heavy episodes of drinking, more of these were unplanned. Some had difficulties to talk about it: ...*I am really bad with words, I get anxious, I get fumbled*. Fears related to disclosing SA including concerns about confidentiality were common, anticipated stigma overreaction, and loss of autonomy...*Everybody just freaks out and wants to get you hospitalized... it doesn´t make me feel better. I don´t want my privileges taken away.*
13. Rogers et al. (2017)	USA	Statistically factor analyses<br>Increase in suicide intent (SI) over the course of hours or days was included.	343 outpatients and 7698 inpatients participated.	Examination of the proposed ASAD (Acute Suicidal Affective Disturbance) in relation to suicidal behavior and existing psychiatric disorders.	The patients had sleep disturbances, marked irritability, social withdrawal, and severe affective states. These factors have the potential to lead an individual with suicidal thoughts to engage in suicide behaviors.<br>ASAD was strongly positively associated with depression, anxiety, rumination, and negative urgency and moderately with cognitive anxiety sensitivity and other facets of impulsivity.
14. Stefenson & Titelman (2016)	Sweden	Quantitatively and qualitatively investigation: medical records, written documentation by care providers, summaries, reports and thematic analysis.	96 completed suicides in one year among in-patients in the health care services were included in the study.	Examination of specific characteristics of suicidal communication and critical events preceding suicide in patients with psychosis. Wasserman´s model of suicidal communication was applied; direct verbal-, non-verbal- and indirect verbal communication.	21 patients of 96 (22%) had a primary diagnosis of psychosis. Three months preceding death, 20 had described one or more negative critical experiences like physical illness, loss of contact with an important other, loss of home, or intolerable side effects of medication. Loneliness, a sense of being abandoned by others, being impoverished, lack of meaningful and daily occupation could not be linked to a specific incident. In most cases it appeared to be culmination of prolonged suffering and suicidal deliberations. 13 had recurrently expressed SI since the first onset of psychosis. 10 reported to have expressed deep despair and hopelessness during the last 4 weeks before. Financial and housing problems were related to shame, worthlessness or exclusion.
15. Søndergaard et al. (2023)	Denmark	Scoping review	28 studies were included	Summarize and dissemination on the lived experience of living with suicidal thoughts.	Many of the articles describe how participants explained living with suicidal thoughts as linked to social experiences in childhood, such as domestic abuse or neglect. Living with suicidal thoughts were also experienced as a loss of sense of oneself. Loss of self-worth were expressed together with loss of meaning in life that could lead to self-hatred and a feeling of worthlessness.
16. Tarrier (2007	United Kingdom	Semi- structured interviews.	35 patients suffering their first episode of psychosis were included in the study.<br>.	Reactions and experiences of psychotic episodes were assessed.	18 had suffered a trauma prior to their psychotic episode. 27 had a persistent loss, change or disruption as a result of their illness. 21 thought that their hopes and aspirations were no longer achievable. 12 had suffered physical harassment or violence, 17 stigma, and 16 social exclusions. 28 felt they had been traumatized by being hospitalized, 23 meant it was severe. They were confused and scared by police insensitivity and had fear of other patients and adverse staff attitudes and being forced to take medication. 13 found aspects of their treatment traumatic, 8 meant it was severe. The criteria of PTSD were met by 12. SA was reported.

Studies included in the review

Authors (year)	Country	Methodology/Design	Participants	Aims	Substance of suffering
17. Trygvadottir et al. (2019)	Island	Phenomenological perspective.	17 interviews were conducted with 7 male survivors.	The experience of male survivors´ suicidal thoughts following sexual violence was investigated.	A strong self-destruction force involved unexpressed unbearable suffering, focused on avoiding frequent thoughts about the violence and finding peace. Thinking of death was a part of everyday life and thoughts about the best way to die by suicide. Hopelessness, lack of self-esteem and loneliness characterized their life. Unexplained physical pain was associated with the violence. Risk behavior like driving too fast without seatbelts, high drinking and consumption of other drugs to kill emotional pain appeared. The self-esteem was shattered. They felt shame, disgusting and worthless.
18. Vásquez and Pandoro (2019)	Spain	Structured interview.	138 women were included.	Suicide attempts (SA) and Stressful Life Events (SLE) among homeless women in Madrid were investigated.	The women suffered from high levels of family and social disengagement, had great difficulties in achieving social/ employment reintegration and significant physical and mental health problems. They described sexual and physical abuse and intimate partner violence. Depression, fearfulness, relationship problems, limited social support, and low self-esteem was strongly associated with SA. 46% of these women had AS.
19. Vatne & Nåden (2012)	Norway	Hermeneutic approach and explorative design.	10 patients at psychiatric wards or by crises resolution teams were interviewed. Nine had experienced one or more SA.	The experiences of persons after suicidal crises or recently completed suicide attempts (SA) were investigated.	Early stages in life affected the self-view; problems and breaks with important people, substance abuse, physical injuries and bullying, excessive work-load, loss of meaningful tasks.... *I have still some of these thoughts, that it is my fault*... Bullying, identity problems and losses like death of a mother and parents´ divorce … *started the blues …I can remember the anxiety…when thoughts about suicide came creeping in over the teens*. They described …*being dragged down by uncomfortable forces … being in a dark room… cannot find the door; don´t find the light switch*. Some felt hopelessness. One told...*I can´t take anymore…I have kept saying this for so long... It has become ever stronger after I became ill*. Rejection, anxiety, pressure and sadness could persist the desire for suicide. For one the SA had to do with rapid mood swings and bipolar disorder. Specific events trigged SA: *Finally, it was too much ... I somehow found no meaning in anything, then I felt: was I worth nothing at all.* One described: *I want to make it an end and at the same time I can see the value of living*. It was a coincidence that many of them were found or managed to report the overdose in time. Life did not change greatly after the attempt. Responsibility in life was difficult and is seen related to the desire to avoid suffering. Losing touch with the world, relationship between suicidal accident and life history, struggling for death and life and feelings of guilt and shame were central. Increasing shame was related to negative attitudes from professionals after SA.
20. Vatne & Nåden (2016)	Norway	Hermeneutic approach and explorative design.	Semi-structured interviews with 10 patients, men and women.	Understanding of patients in the aftermath of suicidal attempts (SA) and finding crucial resources that strengthen the desire to live.	Hopelessness and unbearable pain are central emotions when developing SI. A sense that one does not belong, the perception of burdensomeness and fearlessness in the face of death are factors that exacerbate suicidality. To think about suicide as a potential way out can be consoling. Psychache is (Shneidman) about despair, loneliness, shame and anxiety. Becoming aware of the desire to live and the experience of connectedness was understood as pertaining to the persons inner resources and someone who cares.
21. Vatne & Nåden (2018)	Norway	Hermeneutic approach and explorative design.	In-depth interviews with 10 patients.	The experiences that inspire hope in the aftermath of suicide attempts.	When unable to endure the pain, it became unbearable. A low degree of belonginess and the feeling of being a burden to others increase the risk of suicide. They were in deep loneliness and did not believe that someone or something could help them. They felt despair, loneliness, anxiety, fear, shame, guilt and loss of hope. Three generated themes: hope through encounters, hope through the atmosphere of wisdom and a ray of hope when taking back responsibility. Through dialogue and cooperation, safety and ability to cope with suffering, thereby hope and will to struggle for life is created. Encounters with health personnel could both alleviate and increase the suffering. Being met with respect and kindness alleviated and moralizing increased their shame.
